# The Role of MUC1 in Gastric Cancer Development

**DOI:** 10.3390/cancers17203331

**Published:** 2025-10-15

**Authors:** Iwona Radziejewska

**Affiliations:** Department of Medical Chemistry, Medical University of Białystok, 15-222 Białystok, Poland; iwona@umb.edu.pl; Tel.: +48-(85)-7485675

**Keywords:** gastric cancer, glycosylation, MUC1, mucins

## Abstract

Gastric cancer (GC) remains the most common malignancy and the main cause of cancer-related death worldwide. Due to its asymptomatic beginning, most gastric cancer cases are diagnosed in the advanced stages, which is connected with poor outcomes. Therefore, there is an urgent need for the development of early preventive and screening strategies for GC. A full understanding of GC pathogenesis, including examining key factors participating in cancer progress, is important. MUC1 is a glycoprotein that is highly overexpressed in most epithelial tumors, including gastric tumors. MUC1 presents a specifically altered glycosylation pattern, which is important for the creation of a cancerous environment. This mucin has been proposed as a biomarker for predicting GC outcomes. This review summarizes the involvement of MUC1 in GC progression.

## Simple Summary

Gastric cancer (GC) is among the leading causes of death worldwide, mainly due to late diagnoses. Thus, an important challenge is identifying sensitive and accurate biomarkers that enable the early diagnosis and treatment of this cancer. One such candidate is MUC1, a transmembrane glycoprotein, which is overexpressed and specifically glycosylated in many cancers. The present review summarizes the role of MUC1 in gastric cancer development.

## 1. Introduction

Cancer is reported to be the second largest cause of death worldwide [1]. It is estimated that without strong action, by 2035, cancer cases will increase by almost 25%, making this disease the main cause of death [2]. Despite many years of studies and drug development efforts, most metastatic cancers remain incurable. The most effective targeted therapies, which induce strong tumor reduction, provide only temporary relief. Moreover, such therapies are still only appropriate for a small group of patients. Tumor immunotherapy, one of the more promising methods of treatment, is also only successful for a few types of carcinomas [3]. Cancer is difficult to treat mainly due to its heterogeneity at the genomic level, not only between cancers but also within the same cancer type in different patients or among individual cancer cells in a single tumor in one patient [4]. The many limitations of the current cancer therapies necessitate looking for more effective, holistic therapies.

The World Health Organization indicates that more than 85% of cancers are environmentally induced [5]. Furthermore, it is estimated that 30–50% of all cancer cases could be prevented by lifestyle changes, like eating more vegetables, fruit, and wholegrains; consuming less red and processed meat; avoiding tobacco and alcohol; being physically active; and maintaining a normal bodyweight [1]. There are reports in which the authors consider cancer an ecological disease rather than as a genetic disorder only [4,5,6]. According to the Darwinian evolution concept of “survival of the fittest”, ecological stress factors, such as altered metabolic, mechanical, and cell communication aspects, are responsible for the acquisition of mutations by cancer cells. Under these specific environmental conditions, cells need to compete with each other for survival, and in such a way the fittest cells can withstand and form genetically distinct cell populations, such as tumors. Thus, the progressive transformation of normal cells to cancer cells could be considered an ecological–evolutionary process [6,7]. Such an approach may provide an innovative framework for our understanding of the complex process of cancer and developing more effective preventive and therapeutic applications for patients with cancer [6].

However, to find and apply more successful therapies to cure cancer, all factors that impact cancer development should be thoroughly examined and understood. One of such factors is MUC1, a glycoprotein commonly expressed in epithelial cells. MUC1 is well-known oncoprotein that is highly overexpressed in many cancers, including gastric cancer. There are reports that indicate its role in multiple steps of carcinogenesis. MUC1 overexpression is associated with more aggressive tumors and poor prognoses [8,9,10]. Recently, this mucin has been proposed to be a potential prognostic biomarker for predicting gastric cancer outcomes [10,11]. The present study aimed to summarize the role of MUC1 mucin in gastric cancer development.

## 2. Gastric Cancer

Gastric cancer (GC), despite its declining incidence, is one of the most aggressive, leading cancers in the world due to its overall high prevalence and high mortality rate [12,13,14]. GC remains the fifth the most common malignancy and the fourth main cause of cancer-related death worldwide, followed by lung, colorectal, and liver cancers. Regardless of its overall decreasing incidence in various countries over the past years, mainly due to advances in preventative, screening, and therapeutic strategies, it still creates serious health problems [12,15]. Moreover, clinicians can anticipate more GC occurrences in the future as a consequence of aging populations [16]. Because of its asymptomatic beginning, most GC cases are diagnosed at advanced stages, which is connected with a poor outcome and limits the clinical application and efficacy of surgery, which is the main method of treatment. Many patients who are diagnosed with disseminated tumors are unable to receive surgery. The 5-year survival rate of patients with GC is estimated to be less than 20% [17,18,19]. The survival rate is much better if there is no regional lymph node involvement [11]. Peritoneal metastasis is frequent in GC and occurs in about one-third of cases at the time of diagnosis [20,21]. The prognosis of patients with advanced GC is remarkably improved by chemotherapy. However, such therapy is connected with drug resistance and high toxicity [22]. It has also reported that immunotherapy has improved the current therapeutic situation [12]. Nowadays, due to the latest advances in high-throughput technologies, including microarrays and next-generation sequencing, new therapeutic strategies are being found, e.g., more targeted drugs for genes and signaling pathways have been tested [23]. The early detection of every cancer is crucial to improve the effectiveness of diagnosis, treatment outcomes, and prognosis. However, clinically validated and specific biomarkers are mostly not accessible for early diagnosis in cases of highly lethal GC. Obtaining biomarkers with clinically approved sensitivity and high accuracy remains a current challenge [10,24].

The pathogenesis of GC is complicated; many risk factors for GC have been discovered. They include *Helicobacter pylori* infection with successive chronic atrophic gastritis, risky health behavior, genetic factors, low consumption of fruit and vegetables, and diets high in nitrates and nitrites [25,26,27,28]. *H. pylori* has been classified as a definite human carcinogen by the IARC based on epidemiological and animal studies [29]. MUC1, with specific carbohydrate antigens, is known to be a ligand for *H. pylori* in the stomach [30]. In many areas, improved living conditions related to economic development have led to reductions in *H. pylori* and GC incidences [31,32,33]. The progression of GC in patients occurs in multiple stages, developing from normal epithelium, chronic gastritis, atrophic gastritis, intestinal metaplasia, and dysplasia to carcinoma [32,34]. The morphological, biological, and molecular features within the same tumor and between patients is highly variable in GC. Due to this, GC is considered a highly heterogeneous disease [35,36]. Approximately 95% of stomach cancers are adenocarcinomas, with two major types: intestinal and diffuse. Other phenotypes of GC include mucosa-associated lymphoid tissue lymphomas, gastrointestinal stromal tumors, and carcinoid tumors [37]. The occurrence of GC differs depending on the region. For example, the occurrence is higher in Asia, South America, and Central Europe; the lowest rates are in North America and Africa [35,38]. Apart from that, the male gender dominates in this pathology [39].

## 3. Mucins

Mucins are highly glycosylated proteins with a heterogeneous diversity of O-glycans. They are mainly synthesized by epithelial cells and provide protection as well as lubrication to the epithelial surfaces [40,41].

Based on their localization and molecular structure, mucins can be divided into two types, namely, secreted mucins and transmembrane (epithelial, membrane-bound) mucins. The secretory mucins, which lack a transmembrane domain, form a protective layer and create a physical barrier against pathogens. On the other hand, membrane-bound mucins are type-I membrane proteins that are kept in the plasma membrane because of the presence of a transmembrane domain, and they have, apart from a protective function, a role in different signaling pathways [42]. Epithelial mucins serve as sensors of outer surroundings via extracellular-domain-mediated ligand joining or as an outcome of altered conformations, which can result from changes in biochemical conditions such as pH, ionic composition, and physical interactions. As a result, signals are carried to the interior of the cell throughout the post-translational alterations of the epithelial mucins’ cytoplasmic tail, like phosphorylation, proteolytic events, and others [43]. Moreover, surface-bound signaling mucins have their circulating products that are of the special importance for cancer [41].

Mucins are encoded by specific mucin genes, *MUCs*, followed by genes that express the order in which the mucin gene is found. The secreted mucins are called MUC2, MUC5AC, MUC5B, MUC6, MUC7, MUC8, and MUC19. As membrane-bound mucins, there are MUC1, MUC3A, MUC3B, MUC4, MUC11-13, MUC15-17, MUC20, MUC21, and MUC22 [40,41,44]. The specific attribute shared by all mucin proteins is the presence of a tandem-repeat structure with high contents of three amino acids: proline, threonine, and serine (PTS domain). This part is highly O-glycosylated via N-acetyl galactosamine O-linkages at threonine and serine residues [42]. N-glycosylation sites are found mainly outside this region [41]. It is well-documented that the alterations in mucins glycosylation are among the main molecular changes associated with oncogenic transformations in the gastric tract [45,46]. Generally, glycans play a pivotal role in protein folding, trafficking, and stability. Apart from that, they participate in many cell functions, such as cell adhesion, signaling, and migration. Moreover, they are able to modulate immune recognition and host–pathogen interplay [45,47]. The abnormal glycosylation and substantial splicing of cancerous mucins may also result in the formation of cancer-specific B- and T-cell epitopes, providing opportunities for vaccine design [40].

Aberrant glycosylation occurs in almost all types of human cancers. Changes in mucin O-glycosylation have revealed critical roles in tumor growth and progression. Such alterations can affect the aggressiveness of tumor cells, involving the ability to spread via circulation and metastasize distantly [14,41,44,47,48,49,50]. One of the main carriers of O-glycans in most tumors, including gastric tumors, is the transmembrane MUC1 mucin.

## 4. MUC1

### 4.1. MUC1’s Structure

**Figure 1:**
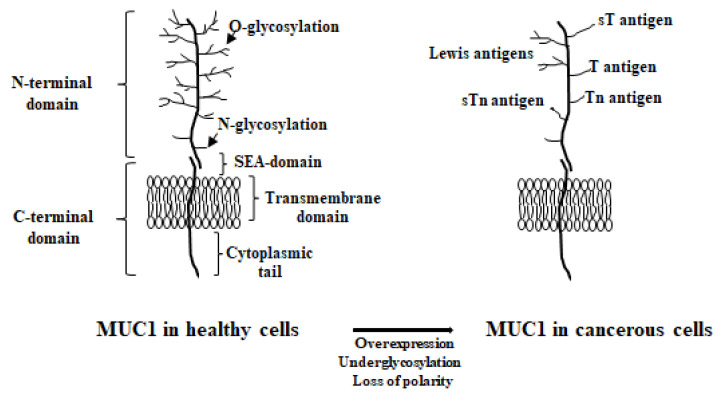
The structure of MUC1 in healthy and cancerous cells.

MUC1, a highly polymorphic glycoprotein with a 120–225 kDa molecular mass, known also as episialin, polymorphic epithelial mucin (PEM), epithelial membrane antigen (EMA), DF3 antigen (recognized by DF3 mAb), and HMFG (human milk fat globule) antigen, is the best-characterized of the transmembrane mucins. *MUC1* is found on human chromosome locus 1q21 and contains seven exons [42,51]. It is a type-I heterodimeric transmembrane glycoprotein containing two main subunits: a large, extensively glycosylated N-terminal domain (MUC1-N) and an intracellular C-terminal domain (MUC1-C). These domains form after MUC1 synthesis, when the molecule is hydrolyzed due to special stress. MUC1-N extends from the cell surface up to 200–500 nm (Figure 1).

Both domains are non-covalently linked through a degenerate sequence [52,53]. The extracellular subunit can be released from the cell surface by proteolytic cleavage and can be found in the circulation. Such dissociation can be stimulated by interferon gamma (INF-γ) and tumor necrosis factor alpha (TNF-α) by activation of specific enzymes involving the matrix metalloprotease ADAM (a disintegrin and metalloprotease)-17 and TNF-α containing enzyme (TACE). The N-terminal domain of MUC1 contains a region of variable numbers of tandem repeats (VNTRs) composed of 20 amino acids (PDTRPAPGSTAPPAHGVTSA), repeated 20–200 times per molecule, and a SEA (sea urchin sperm protein enterokinase and agrin) region [54]. The VNTR region may be highly polymorphic in length and is poorly conserved. The SEA domain is highly conserved and consists of approximately 100 amino acids residues lying close to the membrane on the luminal side. This domain is autoproteolytically cleaved, and after splitting, the extracellular domain is released to the circulation [42]. The peptide backbone formed by the VNTR region provides the scaffold for glycosylation. Glycans are mainly linked to Ser/Thr on the polypeptide backbone by O-glycosidic bonds [53]. The extensively branched O-glycans comprise various monosaccharides such as N-acetyl galactosamine (GalNAc), galactose (Gal), N-acetylglucosamine (GlcNAc), N-acetylneuraminic acid (sialic acid, Nau5Ac), and fucose (Fuc) [44,52,55]. In N-glycans, the amidic nitrogen of asparagine from the polypeptide backbone links to GlcNAc at five possible sites: four in the MUC1-N domain and one in the extracellular region of the MUC1-C domain. N-glycan oligosaccharide chains comprise mannose (Man), xylose (Xyl), and 12 other common monosaccharides [56]. The MUC1-C subunit contains a short extracellular domain of 58 amino acids, a transmembrane part of 28 amino acids, and a cytoplasmic tail of 72 amino acids [42,54,57] (Figure 1).

**Figure 2:**
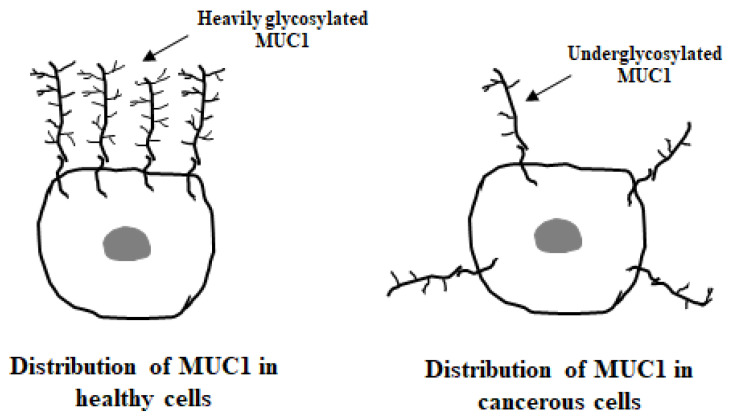
The distribution of MUC1 in healthy and cancerous cells.

In normal cells, MUC1 is located at the apical surface of epithelial cells. In the cancerous state, this mucin is highly overexpressed, up to 10-fold, and loses its cell polarity. It is redistributed over the whole cell surface and within the cytoplasm (Figure 2).

Additionally, in cancers, MUC1 glycosylation is reduced and altered, and specifically modified glycoforms are formed [57,58]. These tumor-associated carbohydrate antigens (TACAs) include GalNAcα-O-Ser/Thr (Tn, Thomsen Nouveau, CD175), Neu5Acα2,6-GalNAcα-O-Ser/Thr (sTn, sialyl Tn, CD175s), Galβ1,3-GalNAcα-O-Ser/Thr (TF, Thomsen–Friedenreich, CD176, T), Neu5Acα2,3-Galβ1,3-GalNAcα-O-Ser/Thr (sTF, sialyl TF) [52]. Most of the presented TACAs are expressed in low amounts in healthy tissues and in increased amounts in cancers [9,59]. Tn and sTn antigens are common especially in breast, prostate, respiratory, pancreatic, ovarian, colon, and gastric tumors [48,55,60,61]. T and sT structures are both expressed in breast cancer, while T antigen is more common in gastric, colon, pancreas, ovary, and prostate malignancies [62,63]. Moreover, in addition to the simple O-glycans present in malignant cells, there are also fucosylated Lewis antigens, such as NeuAcα2,3-Galβ1,3-(Fucα1,4)-GlcNac-R (sialyl Lewis a, sLe^a^,) and NeuAcα2,3-Galβ1,3-(Fucα1,3)-GlcNac-R (sialyl Lewis x sLe^x^,) [52,55,62]. Sialyl Lewis(a) antigen is typical in colon, gastric, pancreas, lung, liver, and breast cancers [52,55], while sialyl Lewis(x) is common in pancreas, gastric, colon, esophagus, ovary, and breast tumors [55]. Very often, the expression of the mentioned sugar structures is correlated with the progression of the disease [47,52].

### 4.2. MUC1’s General Function

MUC1 plays different roles in normal tissues and tumors. In the healthy state, highly glycosylated MUC1 forms a protective barrier and provides protection to the underlying epithelia from extreme environments. The extended, negatively charged carbohydrate branches of the MUC1-N domain create a physical barrier, confer anti-adhesive properties, block cell–cell and cell–extracellular matrix interactions, reduce accessibility to the epithelium, and prevent pathogenic colonization. Moreover, sugar chains oligomerize and form a mucinous gel that lubricates, hydrates, and protects the epithelium from changes in pH, pollutants, and microbes. It has been reported that MUC1 plays a crucial role in *Helicobacter pylori*-associated gastritis via acting as a protective physical barrier against pathogens [8,64,65]. MUC1 is also involved in suppressing the inflammation induced by *H. pylori* [66]. The SEA domain helps regulate cell shedding and adhesion, inhibits the immune response through receptor shielding, and acts as a decoy receptor for invading pathogens [9,53,67,68,69,70]. Apart from these, MUC1 participates in repairing damaged epithelium via mechanisms like epigenetic reprograming and epithelial–mesenchymal transition (EMT). Thus, this mucin maintains the stability and integrity of the epithelial layer [57,71].

Contrary to its protective function in healthy epithelial cells, in cancer tissues, MUC1 overexpression, together with reduced glycosylation and altered polar distribution, has a different function. Cancerous MUC1 participates in some key processes involving inflammation, proliferation, migration, EMT, and epigenetic reprogramming [72,73,74,75,76]. Thus, MUC1 is considered an oncogenic driver as it increases aggressiveness and metastatic potential [57,72,77,78]. Moreover, this mucin reduces the drug-induced release of mitochondrial pro-apoptotic factors and attenuates apoptosis [43,79]. The MUC1-C domain is involved in transducing signals and regulating cell growth, differentiation, and survival signals. It can form complexes with transcription factors and translocates to the nucleus, where it may influence gene transcription. Moreover, it simplifies immune evasion via interactions with immune cells in the tumor microenvironment [80]. Apart from that, the extracellular O-glycosylated domain can be released and found in the circulation, which may interfere with mucin immunotherapy [81].

## 5. MUC1 in Gastric Cancer

### 5.1. MUC1 Localization

In the healthy condition, the human stomach expresses epithelial MUC1 and two secretory mucins: MUC5AC and MUC6. Additionally, in the cancerous state, the intestinal secretory MUC2 mucin is present [82]. MUC1 protein is observed t the surface foveolar cells in the whole stomach, in mucosal neck cells, and the chief cells of the gastric fundus and antrum, as well as in the pyloric gland, commonly by staining the apical side of the cell membrane and acting diffusely in the cytoplasm [83,84]. One of the crucial molecular events occurring during gastric carcinogenesis is the loss of the well-defined mucin expression pattern, which is observed in the normal epithelium.

### 5.2. MUC1 Action in GC

Ohno et al. [84] indicated that MUC1-positive expression is a marker of malignancy in advanced GC. The authors noticed the role of the interactions between MUC1 and cell adhesion molecules, such as E-cadherin, in epithelial tissue integrity. E-cadherin is a calcium-dependent molecule that works as a tumor-inhibitory factor. The loss of E-cadherin-mediated cell–cell adhesion is prerequisite for tumor cell invasion and metastasis formation [85]. MUC1 stimulates tumor invasion via the attenuation of E-cadherin. As the result of multivariate analysis, the authors stated that patients with abnormal E-cadherin/MUC1-positive expression were independently and significantly correlated with a poor prognosis and had a relative risk of death, which was 2.8 times higher than that of other patients. Similar findings were presented by Zhang et al. [82] and Tanaka et al. [86], who revealed that MUC1 may interfere with the function of E-cadherin and has a synergic effect on the progression of GC.

It was reported that the length of the VNTR allele of the *MUC1* gene affects the patient’s sensitivity to *H. pylori* [65,87]. The smaller size of MUC1 VNTR alleles may be correlated with *H. pylori* infection and a higher possibility of GC development [88]. *H. pylori* binding to gastric mucosal epithelial cells induces inflammatory responses, gastric epithelial cell mutation, inhibition of apoptosis, stimulation of angiogenesis, and cell proliferation [89,90]. In GC cells, MUC1 interacts with the *H. pylori*-cytotoxin-associated gene A (*cagA*), a main bacterial virulence factor; upregulates Wnt–β-catenin signaling; and increases cyclin-D1-dependent cell proliferation, all of which contribute to gastric carcinogenesis [91]. It was also shown that *H. pylori* upregulates the MUC1 expression in GC cells through STAT3 and CpG hypomethylation [92]. On the other hand, the potential of MUC1 to reduce *H. pylori* colonization was demonstrated, which was related to MUC1 acting as a releasable decoy, because bacterium binding to MUC1 on the epithelial cells triggers the release of the extracellular domain of this mucin, effectively shedding *H. pylori* from the mucosal surface, consequently limiting its ability to adhere to the gastric epithelium [93]. Moreover, MUC1 suppresses the IL-1β secretion by macrophages in response to *H. pylori* and thus protects against the development of atrophic gastritis, a step on the path to GC [94]. IL-1β secretion in response to *H. pylori* occurs via the NLRP3 inflammasome, which is required for NF-kB activation [95]. MUC1 was revealed to attenuate the NLRP3 inflammasome by inhibiting the activation of TLR signaling during the initial steps [87,94].

Aberrant glycosylation, appearing as TACAs on MUC1, is a common phenomenon associated with oncogenic transformation. It was revealed that polymorphism in the MUC1 tandem repeats influences the expression of specific cancer sugar antigens in GC cells and may therefore allow the identification of subgroups of patients that develop more aggressive tumors expressing T antigen [96]. Authors demonstrated a statistically significant correlation of the occurrence of this antigen with large tandem-repeat alleles of MUC1. Epitopes such as T antigen are crucial in the cell invasion or metastatic potential of many epithelial cancers as they mediate the interactions between cells and the endothelium. Numerous reports have demonstrated that the interactions of Gal-3 with MUC1 via T antigen are essential for metastatic cell adhesion in cancer progression [44,52,97]. Recently, Bao et al. [14] proposed C1GalT1, an enzyme responsible for T-antigen formation, as an independent factor of poor prognoses in patients with GC. The authors revealed that C1GalT1-mediated O-glycan T-antigen increases promoted GC cell migration and invasion, which could be due to C1GalT1 targeting the T antigen of MUC1. According to this, C1GalT1 was suggested as a novel therapeutic target for GC treatment. In another study, Fujii et al. [98] revealed that enhancing αGlcNAc biosynthesis on MUC1 binding protein attenuates the proliferation, motility, and invasiveness of GC cells. The authors suggested that αGlcNAc acts as a tumor suppressor by inhibiting MUC1 signaling.

The trefoil factor family (TFF) is a secreted protein family, which is composed of TFF1, TFF2, and TFF3 and characterized by the trefoil domain. In the cancer state, these proteins are involved in numerous biological processes, including cell proliferation and apoptosis. TFF1 is a gastric-related tumor suppressor gene, and TFF2, present in gastric mucosa, triggers cell migration signaling to promote epithelial repair [99]. TFF2 expression is frequently silenced in GC [100]. It has also been reported that epithelial cells synthesize TFF peptides together with mucins, and both factors play crucial roles in protecting the mucosal epithelial cells from a variety of insults [101,102]. Interesting results on the association between TFF peptides and MUC1 in GC were reported by Ge et al. [32]. The authors revealed that MUC1 negatively correlated with the methylation of TFF2 and positively regulated the TFF2 expression in GC. Thus, the authors shed new light on the strong connection between MUC1 and TFF2 regulation. They also indicated that patients with a low expression of MUC1 or TFF2 had a worse outcome, indicating that these factors act as prognostic biomarkers in GC.

Deng et al. [103] revealed that MUC1 is a direct target of miR-206 in GC. There was an inverse correlation between the MUC1 mRNA level and miR-206 expression. The authors found that MUC1 overexpression reverses the suppression of miR-206 in cell proliferation and metastasis as well as the induction of miR-206 on apoptosis in GC cells. The authors concluded that the antitumor activities of miR-206 on GC cells are partially associated with its inactivation of MUC1. Moreover, other authors showed that MUC1 induced trastuzumab resistance in HER2-positive GC cells [104].

**Figure 3:**
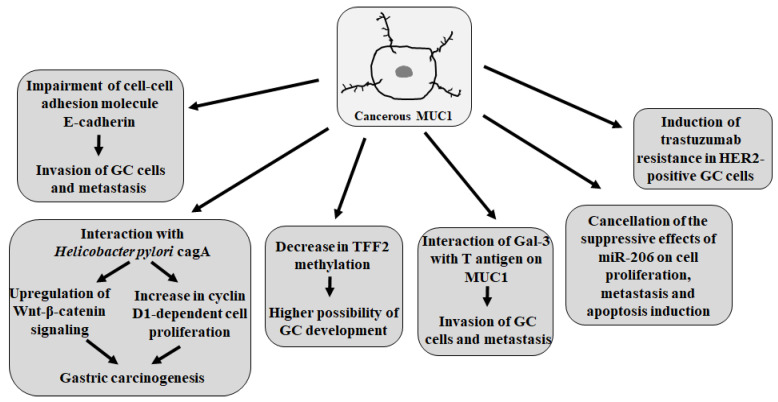
Main functions of MUC1 in GC.

The main functions of MUC1 in GC are presented in Figure 3.

### 5.3. VNTR Polymorphism of MUC1 Gene and the Risk of Stomach Cancer

There are studies revealing the correlation between the VNTR polymorphism of MUC1 and the risk of GC. It was demonstrated by studies in a Portuguese population that the smaller MUC1 VNTR alleles are associated with an increased risk of gastric carcinoma as well as of chronic atrophic gastritis [105]. In addition, Jia et al. [29] provided evidence that some common variations in MUC1 genes contribute to an increased probability of stomach cancer. Several polymorphisms in the MUC1 are reported in the literature. Among them, MUC1 rs4072037 is one of the commonly investigated [30]. Liu et al. [106] reported an association between variant alleles and GC risk: the G allele was significantly associated with a decreased risk of GC in Asian populations, regardless of the anatomic location or pathological subtype, compared with the A allele. Similar results were obtained in another study, where the authors also revealed that the MUC1 rs4072037 polymorphism reduced the risk of GC [107]. Giraldi et al. [65] also confirmed the protective effect of the MUC1 rs4072037 polymorphism on the risk of GC under a dominant model. However, there were also findings among patients in northern Iran that indicated that the rs4072037G>A polymorphism interacts with *H. pylori* infection to increase the risk of GC [108]. Apart from that, genome-wide association studies (GWASs) have identified several risk loci located in the *MUC1* gene that are significantly correlated with susceptibility to GC [32,109,110,111].

### 5.4. MUC1 as Prognostic Factor

As mentioned above, MUC1 is highly overexpressed in most epithelial cancers, including GC, which is associated with poor patient prognoses [41,112,113,114]. MUC1 is an indicator of clinicopathological significance in GC. In pathological conditions, MUC1 changes its expression method and rate [11,83]. Moreover, MUC1 has been identified as a genetic marker that is significantly associated with GC susceptibility, which emphasizes its potential as a diagnostic and prognostic tool [115,116]. Some clinical reports have revealed that the prognosis of patients with GC depends on the tumor’s histological morphology and the tumor node metastasis (TNM) classification [9,115,116]. Recently, Kim et al. [10] reported a prognostic role of MUC1 in patients with GC in the U.S. considering TNM staging. The study showed that this mucin’s expression significantly correlated with the pathological features of a poor prognosis. Patients with MUC1 overexpression had a higher rate of deeper tumor depth (T staging), lymph node metastasis (N staging), and distant metastasis (M staging). Thus, the authors revealed that MUC1 expression was associated with aggressive pathological features, and they suggested MUC1 as a useful prognostic marker for predicting cancer outcomes. Moreover, subgroup analysis in patients with early GC showed that the proportion of early cancer categorized as non-curative resection was two-fold higher in patients with a high level of MUC1 expression compared to those without high MUC1 expression. Apart from that, a significant association was found between high MUC1 expression and lymphovascular invasion in patients with early GC. Similar results were presented by Duraes et al. [117], who reported that MUC1-positive patients with GC had a higher rate of vascular invasion and lymph mode metastasis. Lee et al. [118] showed that the MUC1-positive patients with GC in Korea had a significantly worse survival rate than those who were MUC1-negative. Moreover, other authors concluded that MUC1 was an independent prognostic factor of GC as they revealed that MUC1 expression correlated with a poor outcome irrespective of its glycosylation level [119]. Other authors pointed out that MUC1 positivity might be correlated with the invasiveness of gastric carcinoma cells, as demonstrated by experiments revealing that MUC1-positive tumors were associated with synchronous liver metastasis [11,120]. In addition, authors showed that MUC1-positive patients with GC displayed a lower 5-year survival rate than patients who were MUC1-negative [11]. In another study, Tamura et al. [121] demonstrated that MUC1 and MUC4 expressions in GC may be related to factors influencing a poor prognosis, such as lymphatic invasion, venous invasion, as well as lymph node metastasis, via different mechanisms. Moreover, Yang [18] revealed that MUC1 as well as MUC5AC, KRT7, GAPDH, and CD44 are related to not only GC but also the apoptosis pathway, suggesting these factors are prognostic biomarkers of GC. Apart from that, Wang et al. [122] reported the increased expression of MUC1 mucin as a marker of differentiation in GC. The authors also demonstrated that both the T and B cells of the immune system are activated by MUC1 peptide epitopes.

### 5.5. MUC1 as Therapeutic Target

Due to its overexpression, specifically altered glycosylation, and participating in different steps of GC development, MUC1 is considered an attractive target in cancer treatment. TACAs of MUC1 have been considered crucial targets for cancer vaccination development [123]. Such glycan-based cancer therapies have been studied especially for prostate and breast cancers [124,125]. Preclinical and clinical research based on MUC1-targeted therapy in GC is still ongoing. Monoclonal antibodies, immunoconjugates, small-molecule peptides, and vaccines are in various stages of studies. Recently, Yu et al. [114] demonstrated that altered peptides from MUC1, such as HLA-A0201-restricted CTL epitopes, could serve as peptide vaccines or constitute components of peptide-loaded dendritic cell vaccines for GC therapy. Some authors have reported that combinations of anti-MUC1 monoclonal antibody with chemotherapeutic agents might also be promising strategies in GC treatment [126,127].

## 6. Conclusions

Based on the presented information, it can be concluded that MUC1 might be a key factor in the development of GC and, due to this, may be an attractive target in various therapeutic strategies. This mucin may be a helpful prognostic biomarker for predicting GC outcomes. Identifying specific markers, including genetic ones, linked to GC offers promising directions for enhancing patient care through providing more personalized medicine, which would result in the early recognition of high-risk individuals, facilitating fast intervention.

## Data Availability

No new data were created or analyzed in this study. Data sharing is not applicable to this article.
